# Understanding the Lived Experience and Bereavement of Caregivers of People with Alzheimer’s Disease: A Mixed-Methods Study Protocol

**DOI:** 10.3390/healthcare14070899

**Published:** 2026-03-31

**Authors:** Nerea Risquez-Salgado, Sara García-Bravo, Elisabet Huertas-Hoyas, Jorge Pérez-Corrales, María Salcedo-Perez-Juana, Madeleine Donovan, Domingo Palacios-Ceña, Elisa Bullón-Benito, Cristina García-Bravo

**Affiliations:** 1 PhD Student Program in Health Sciences, Universidad Rey Juan Carlos, 28922 Alcorcón, Spain; n.risquez.2020@alumnos.urjc.es; 2 Research Group of Participation, Roles, Occupations and Activities for Community Transformation (PROACT), Department of Physical Therapy, Occupational Therapy, Rehabilitation and Physical Medicine, Universidad Rey Juan Carlos, 28922 Alcorcón, Spain; elisabet.huertas@urjc.es (E.H.-H.); cristina.bravo@urjc.es (C.G.-B.); 3 Research Group of Humanities and Qualitative Research in Health Science (Hum&QRinHS), Department of Physical Therapy, Occupational Therapy, Rehabilitation and Physical Medicine, Universidad Rey Juan Carlos, 28922 Alcorcón, Spain; jorge.perez@urjc.es (J.P.-C.); maria.perezjuana@urjc.es (M.S.-P.-J.); domingo.palacios@urjc.es (D.P.-C.); 4 Independent Researcher, 13409 Berlin, Germany; madeleine.c.donovan@gmail.com; 5 Department of Physical Therapy, Occupational Therapy, Rehabilitation and Physical Medicine, Universidad Rey Juan Carlos, 28922 Alcorcón, Spain; elisa.bullon@urjc.es

**Keywords:** Alzheimer’s disease, family caregivers, caregiver burden, quality of life, occupational balance, mixed methods, qualitative interviews, cross-sectional study

## Abstract

**Highlights:**

**What are the main findings?**

- Caregiving in Alzheimer’s disease is experienced as a continuum of relational, occupational, and identity-related losses that begins during active care and extends into bereavement.
- A convergent mixed-methods approach allows the integration of caregiver burden, health-related quality of life, and occupational balance with in-depth narratives of adaptation and meaning-making.

**What are the implications of the main findings?**

- Publishing the protocol promotes transparency, reproducibility, and methodological transferability to other caregiving contexts and neurodegenerative conditions.
- The protocol facilitates the development and future evaluation of holistic, occupation-centered interventions adaptable across care settings and stages of the disease.

**Abstract:**

**Background**: Alzheimer’s disease (AD) is a progressive neurodegenerative disorder that severely affects cognitive, behavioral, and functional abilities, creating a substantial burden for family members who provide continuous care. Caregivers often experience role changes, occupational imbalance, emotional distress, and reduced quality of life, although some report personal growth. These experiences extend beyond active caregiving and include anticipatory grief during disease progression and grief after the relative’s death. Despite this continuum, few studies have examined caregiving, loss, and bereavement from an integrative perspective. This protocol describes a mixed-methods study aimed at exploring the lived experiences of family caregivers of individuals with AD, focusing on how evolving relational, occupational, and identity-related losses influence their well-being and adaptation. **Methods**: A parallel convergent mixed-methods design will be used. The quantitative component consists of a cross-sectional observational study including 66 caregivers recruited through purposive sampling across kinship categories (spouse/partner, adult child, grandchild) and care settings (home care with day-center attendance vs. institutionalized care). Data will be collected using the Zarit Burden Interview, Role Checklist, Short Form-36 Health Survey, and Occupational Balance Questionnaire. Descriptive and subgroup analyses will be conducted using SPSS (version 27). The qualitative component comprises a multiple-case study with approximately 36 participants across three groups: caregivers living with individuals with AD, caregivers of institutionalized relatives, and bereaved family members. Semi-structured interviews (45–80 min) will be conducted online or in person, transcribed verbatim, and analyzed thematically using MAXQDA (version 26). Integration will follow a concurrent approach, combining quantitative and qualitative results through joint narratives and displays to produce a comprehensive interpretation. **Discussion**: This study aims to deepen understanding of the caregiving–grief continuum in families affected by AD by integrating quantitative indicators of burden, health status, and occupational balance with qualitative accounts of adaptation and meaning-making. Findings are expected to support the development of holistic, evidence-based interventions that promote caregiver well-being throughout the care trajectory and during bereavement. **Ethics and Dissemination**: Ethical approval was granted by the Research Ethics Committee of Universidad Rey Juan Carlos (Code: 041220246522024; 15 October 2025). ClinicalTrials.gov Identifier: NCT07251738. Registered November 2025. Protocol version: Version 2.

## 1. Introduction

Alzheimer’s disease (AD) is defined as a progressive neurodegenerative disorder characterized by deficits in memory, language, and behavior [1]. It is a multifactorial disease of largely unknown etiology, with only a small proportion of cases having a clear genetic origin [2]. AD is the most common cause of dementia and leads to progressive neuronal damage in brain regions responsible for higher cognitive functions, affecting memory, thinking, language, and behavior [3].

According to the World Health Organization (WHO), more than 55 million people worldwide currently live with dementia, and this figure is expected to exceed 110 million by 2050. AD is also one of the leading causes of disability and dependence among older people, resulting in a gradual loss of autonomy and a significant impact on Activities of Daily Living (ADL), as well as on social and family relationships [4].

Given the limited effectiveness of pharmacological treatments, current care strategies increasingly focus on non-pharmacological interventions, such as cognitive stimulation, ADL training, and adapted physical activity, aimed at maintaining functionality and quality of life [5,6]. However, the progressive nature of the disease places a substantial burden on the family environment, where most care is provided.

In this study, the term caregiver refers to a family member who assumes primary responsibility for the care of a person with AD, providing ongoing physical, emotional, and social support, typically without financial compensation [7]. This role requires continuous adaptation and often entails a profound reorganization of daily life, including changes in routines, relationships, and personal priorities [8].

Previous research has shown that caregivers of people with AD are predominantly women, often spouses or daughters, who provide long-term, informal care [9,10]. This sustained commitment is associated with significant physical, psychological, and social consequences, including increased levels of caregiver burden, depressive symptoms, anxiety, sleep disturbances, and reduced quality of life [11,12]. In addition, caregiving frequently affects occupational balance, as caregivers struggle to maintain a satisfactory distribution of time and engagement across meaningful activities, including self-care, productivity, and leisure [13,14].

Nevertheless, caregiving is not exclusively associated with negative outcomes. Some caregivers report positive aspects, such as personal growth, strengthened emotional bonds, and the development of resilience and empathy [15]. However, as the disease progresses, caregivers often experience a series of cumulative and progressive losses, including changes in the identity and relationship with the person with AD, loss of shared roles, and disruption of future expectations.

In this context, increasing attention has been paid to the concept of anticipatory grief, understood as the emotional response to the expected loss that occurs before the death of a loved one. In dementia, this process may begin early in the disease trajectory and intensify as cognitive and functional decline progresses. Caregiving and bereavement are therefore not separate processes but form a continuum, in which caregivers experience ongoing losses before death and continue to process grief after bereavement [16,17,18].

Despite growing recognition of caregiver burden, fewer studies have explored this caregiving–bereavement continuum from a comprehensive perspective, integrating both the day-to-day caregiving experience and the grieving process before and after death. In particular, there is limited research examining how these experiences affect occupational balance, identity, and overall well-being.

Understanding caregiving as a dynamic and multidimensional process, from the assumption of the caregiving role to bereavement, is essential for designing effective support strategies. Given the complexity of this phenomenon, mixed-method approaches are particularly suitable, as they allow the integration of quantitative measures (e.g., burden, quality of life, occupational balance) with qualitative insights into caregivers’ lived experiences and meanings.

Therefore, this study protocol proposes a mixed-methods design aimed at comprehensively examining the life experience of caregivers of people with AD, with a particular focus on how cumulative losses (relational, occupational, and existential) influence their well-being, sense of identity, and adaptation. This protocol describes the theoretical and methodological frameworks, data collection procedures, recruitment strategies, integrated analysis plan, and ethical considerations. The findings are expected to contribute to the development of holistic support interventions that address caregivers’ needs throughout the caregiving and bereavement continuum.

### 1.1. Objective of the Study

The aim of this study is to comprehensively examine the impact of caring for a person with AD on caregivers’ daily lives using a mixed-methods approach by assessing caregiving burden, occupational roles, occupational balance, quality of life, and grief experiences.

The specific objectives are as follows:

#### 1.1.1. Quantitative Objectives

Objective 1: To analyse caregiving burden and changes in occupational roles among family members and/or caregivers of people with AD.

Objective 2: To assess the quality of life of family members and/or caregivers of people with AD.

Objective 3: To evaluate occupational balance among family members and/or caregivers of people with AD.

Objective 4: To compare caregiving burden, occupational roles, quality of life, and occupational balance between caregivers of people with AD who are institutionalized in nursing homes and those living at home.

#### 1.1.2. Qualitative Objectives

Objective 5: To explore caregivers’ lived experiences and perceptions regarding the impact of caring for a person with AD on their daily life and occupational participation.

Objective 6: To explore caregivers’ experiences of anticipatory grief and bereavement associated with the progression and loss of a person with AD.

#### 1.1.3. Mixed-Methods Integration Objectives

Objective 7: To integrate quantitative and qualitative findings in order to obtain a comprehensive understanding of how caregiving context and experiences shape caregivers’ occupational lives and well-being.

Although the quantitative component of this study is primarily exploratory, a set of a priori hypotheses is proposed to guide the analysis in line with the study objectives:

It is hypothesized that caregivers of people with AD living at home will report higher levels of caregiving burden, greater disruption in occupational roles, lower quality of life, and poorer occupational balance compared to those whose relatives are institutionalized (Objective 4).

In addition, it is expected that caregivers with closer family relationships (e.g., spouses) will present higher levels of emotional burden and greater alterations in occupational roles and balance compared to other family members (Objectives 1 and 3).

Finally, it is hypothesized that higher levels of caregiving burden will be associated with lower quality of life and poorer occupational balance (Objectives 1–3).

## 2. Materials and Methods

### 2.1. Study Design

For this project, a mixed-methods parallel convergent study will be designed. The purpose of mixed-methods research is to provide comprehensive and contextualized answers to questions raised in the health sciences, integrating different approaches to compensate for the limitations inherent in a single method or methodological perspective, through the parallel or simultaneous collection and analysis of qualitative and quantitative data [15].

A convergent design is particularly suitable for this study because the impact of caring for a person with AD involves both measurable outcomes and complex subjective experiences. Quantitative methods allow the assessment of caregiving burden, occupational balance, role performance, and quality of life, while qualitative approaches enable the exploration of caregivers’ lived experiences, perceptions of caregiving, and grief processes. The use of a convergent design will allow both types of data to be collected during the same phase of the study, analysed independently, and subsequently integrated to provide a more comprehensive understanding of caregivers’ experiences [15].

To this end, a quantitative observational cross-sectional study (QUAN) and a qualitative case study (QUAL) will be designed, with data collection and analysis for both methods carried out simultaneously. Qualitative case studies offer the opportunity to investigate in depth different perspectives of a specific health and disease phenomenon in its real context, using the perspective of people in their own social environment [19]. Cross-sectional studies, being observational in nature, offer a snapshot of the characteristics of subjects at a given point in time. However, they lack a follow-up period, which prevents the establishment of cause-and-effect relationships [20].

Integration of the quantitative and qualitative strands will take place during the interpretation phase of the study. Quantitative findings will provide measurable information about caregiving burden, occupational roles, occupational balance, and quality of life across caregiving contexts, while qualitative findings will offer deeper insights into caregivers’ experiences and the meaning attributed to caregiving and grief. The integration of both datasets will allow identification of areas of convergence, complementarity, or divergence between results, contributing to a more comprehensive understanding of how caregiving context and personal experiences shape caregivers’ occupational lives and well-being.

The development of this study protocol followed the SPIRIT 2025 guidelines [21]. Supplementary Material S1. The guidelines of the National Institutes of Health for mixed-methods research in health sciences [22] will be adopted for the development of this project. Likewise, the STROBE recommendations [23] will be followed for the observational study (QUAN), while the SRQR [24] and COREQ [25] guidelines will be followed for the qualitative design (QUAL).

The QUAN will focus on assessing occupational balance, role performance, caregiving overload, and quality of life among caregivers or family members of people with AD. To this end, two analysis groups will be established: one composed of people who live with family members with AD at home but attend a day center, and another composed of caregivers of family members institutionalized in a nursing home located in the Community of Madrid. Each group will be divided into three subgroups, depending on the family relationship to the person with AD (husband/wife, son/daughter, and grandson/granddaughter).

The QUAL will focus on exploring the experiences of family members/caregivers of people with AD with regard to caregiving and its impact on daily life. As in the QUAN design, two groups of participants will be established: family members who live with people with AD and family members who have institutionalized relatives with AD. In order to explore experiences in different family contexts and existing generational differences, these groups will be subdivided into three groups each to try to include different family roles (husband/wife, son/daughter, and grandson/granddaughter) in the study. In addition, a third group will be included to learn about the experiences of family members who have experienced the loss of a relative with AD.

**Figure 1:**
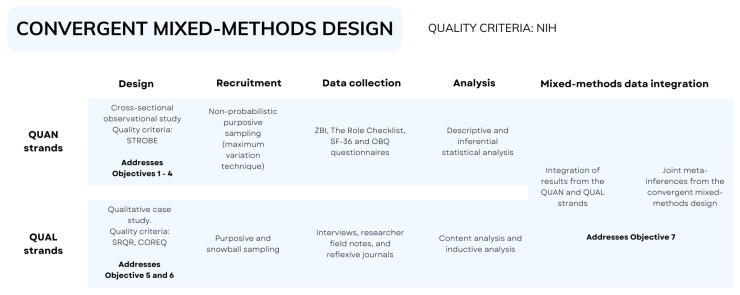
Convergent parallel mixed-methods design.

Figure 1 illustrates the convergent parallel mixed-methods design used in this study. Quantitative and qualitative data will be collected during the same phase, analysed independently, and subsequently integrated during the interpretation stage.

### 2.2. Sample/Participants

#### 2.2.1. Sampling Strategy and Number of Participants Included

For the development of this project, non-probabilistic purposive sampling will be used, based on the maximum variation technique [26,27]. This strategy allows participants to be deliberately selected to represent the greatest possible diversity within the phenomenon under study, with the aim of capturing different perspectives and experiences. In this case, variation will be ensured based on the different family roles played by caregivers and the institutionalization or home care status of the person with AD [26].

In qualitative studies, there is no formula for calculating sample size in advance, as there is no intention to extrapolate results or calculate statistical significance [27,28]. However, different authors consider multiple approaches and criteria to substantiate and determine sample size [29,30]. In the case of a multiple-case study, it is recommended to work with at least 2 to 4 cases to allow for comparison between them and enrich the analysis with different perspectives [31,32]. According to the authors, multiple case studies provide greater robustness to the analysis of the phenomenon than a single case study, as they allow different contexts and conditions to be explored, facilitating comparisons between them and increasing the credibility of the results obtained [31,32]. Therefore, for the present study, there will be a sample of 12 participants per group. Since there are three groups in the QUAL design, there will be a total of 36 participants.

In the QUAN design, the sample size was calculated using the G*Power program (version 3.1; one-factor ANOVA, three groups, 1:1:1 allocation) with a significance level (α) of 0.05 and a statistical power (1 − β) of 0.80. To detect a minimum difference of 12.62 units between groups, the result was 17 participants per group (n = 51). Considering a potential loss rate of 20%, the required recruitment size was adjusted to n = 51/(1 − 0.20) = 63.75. After rounding up and in order to maintain balanced groups, a total of 66 participants (22 per group) will be recruited. The sample size calculation was performed to ensure sufficient statistical power for group comparisons in the quantitative component of the study. Given the expected size of some subgroups, analyses involving subgroup comparisons will be interpreted with caution and considered exploratory.

#### 2.2.2. Inclusion Criteria

To carry out this study, three groups of relatives of people diagnosed with AD will be formed.

*Inclusion criteria for Group 1:* relatives of people diagnosed with AD by a neurologist or geriatrician; who have their relative institutionalized in a nursing home; who agree to participate voluntarily in the project; and who have signed the informed consent form.

*Inclusion criteria for Group 2:* relatives of people diagnosed with AD by a neurologist or geriatrician; who live with and/or care for the person with AD; who attend a Day Center or a rehabilitation center; who agree to participate voluntarily in the project; and who have signed the informed consent form.

*Inclusion criteria for Group 3:* relatives of people diagnosed with AD by a neurologist or geriatrician; who have suffered the loss of a relative with AD; who have lived with and/or cared for the person with AD until the end of their life; who agree to participate voluntarily in the project; and who have signed the informed consent form.

In addition, after accepting participation in the QUAN design, participants will be offered the opportunity to participate in the QUAL design. For the subdivision of the groups, participating family members will be divided based on kinship:

- Subgroup 1: husband or wife.
- Subgroup 2: daughter or son.
- Subgroup 3: grandson or granddaughter.

These case groups have been selected based on the different roles assumed by various family members, with the aim of exploring experiences in different family contexts and existing generational differences.

#### 2.2.3. Exclusion Criteria

Individuals who have not lived with or cared for a relative with AD or who do not agree to participate voluntarily in the study will be excluded. In addition, individuals with cognitive impairment or communication difficulties that may prevent participation in the interviews, those presenting severe emotional distress at the time of recruitment, and those unable to provide sufficient caregiving experience will be excluded. In the case of bereaved participants, individuals who have experienced a very recent loss may also be excluded to minimize the risk of acute emotional distress. These criteria are established to ensure participant safety and data quality.

All participants will be required to provide written informed consent prior to participation.

### 2.3. Recruitment

Participants will be recruited in a residential care home and a day care center. To this end, the principal investigator will hold meetings to explain what the project consists of, the different phases, and the various data collection tools. After this, those who express an interest in participating in the study will be contacted again to confirm their participation and sign the informed consent form.

For Group 3 (interviews with relatives who have lost a family member with AD), participants will be recruited through direct contact with a relative of a person with AD who has recently died and who was institutionalized in the residence where the sample will be recruited. Through this contact, an attempt will be made to recruit other family members who have suffered the loss of a person with AD. To this end, the principal investigator will hold meetings with participants who express their willingness to participate, in order to explain what the project consists of, the different phases, and the different data collection instruments. After this, those who express an interest in participating in the study will be contacted again to confirm their participation and sign the informed consent form.

### 2.4. Instruments/Measures/Assessment Scales

**Figure 2:**
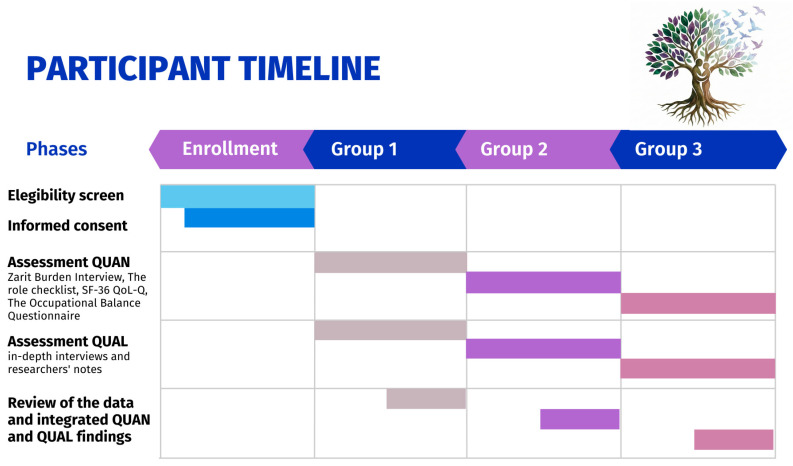
Participant Timeline.

For this project, various phases of data collection will be carried out with participants using different data collection instruments. The participant timeline is presented in Figure 2. Among the instruments to be used in the QUAN design are the following:

- Zarit Burden Interview (ZBI): This tool is designed to assess the level of perceived burden experienced by informal caregivers of dependent individuals. It consists of 22 items that explore emotional, social, and physical aspects related to caregiving, allowing for the identification of the impact of the caregiving role on quality of life. Each item is scored on a Likert scale, with higher scores indicating greater burden [33,34]. The ZBI has demonstrated good internal consistency (Cronbach’s α > 0.80) and has been widely validated in caregiver populations, including caregivers of people with dementia [33,34,35,36].
- The Role Checklist: A tool based on the Model of Human Occupation (MOHO) designed to assess the occupational roles that a person performs throughout their life. It allows current, past, and future roles to be identified, as well as the subjective value assigned to each one. This tool is useful for understanding how occupational roles contribute to a sense of identity, the organization of daily life, and overall well-being, facilitating the planning of client-centered interventions [37,38]. Previous studies have supported its content validity and clinical utility in occupational therapy contexts [37,38,39].
- The SF-36 (Short Form-36 Health Survey Quality of Life Questionnaire): A widely used tool for measuring health-related quality of life. It assesses eight dimensions: physical functioning, physical role, bodily pain, general health, vitality, social functioning, emotional role, and mental health. It is a generic instrument, applicable to diverse populations and useful in both research and practice, providing a broad profile of people’s health status [40,41]. It is a instrument widely validated internationally, showing good reliability (Cronbach’s α typically > 0.80 across domains) and construct validity [40,41,42].
- The OBQ (Occupational Balance Questionnaire): An assessment tool designed to measure occupational balance, understood as people’s perception of the adequate distribution of their time and energy in meaningful activities. This questionnaire explores aspects related to satisfaction and management of daily occupations, considering both the quantity and quality of the activities performed. It has proven useful in clinical and research contexts to identify occupational imbalances that can affect health and well-being. The original version was developed in Sweden and has been adapted to various languages and cultures, maintaining its validity and reliability [43]. The OBQ has shown good internal consistency (Cronbach’s α ≈ 0.87–0.90) and adequate validity in the Spanish population, supporting its use in research and clinical contexts [43,44].

In addition to the standardized instruments, sociodemographic and caregiving-related variables will be collected, including age, sex, education level, income, duration of caregiving, relationship to the person with AD, and stage of the disease, in order to better characterize the sample and contextualize the quantitative findings.

The instruments to be used in the QUAL design will be in-depth interviews and researchers’ notes taken during data collection [45].

Data collection using semi-structured in-depth interviews consists of asking open-ended questions to allow participants to narrate what is most relevant from their perspective and experience (Table 1, Table 2 and Table 3). The in-depth interview will last between 45 and 80 min. An open-ended question guide will be used to conduct the interviews to avoid the researchers steering the participants [45,46].

The semi-structured interview guide is organized into thematic blocks designed to explore key aspects of the caregiving experience, including the diagnosis process, daily caregiving before and/or after institutionalization, the perceived impact on participants’ lives and, where applicable, experiences of anticipatory grief and bereavement. Each block is aligned with the study objectives and aims to facilitate in-depth exploration of participants’ experiences from their own perspectives.

The interview guide will be used flexibly. Not all questions will necessarily be asked in every interview, as the interviewer will adapt the sequence and depth of questions according to participants’ responses and the flow of the conversation. Participants will be informed that they may decline to answer any question or stop the interview at any time.

To minimize recall bias in bereaved participants, interview questions will focus on specific caregiving periods and concrete experiences, and participants will be encouraged to provide detailed accounts of events rather than general reflections.

Interviews will preferably be conducted via the Teams platform (Microsoft). Interviews will be recorded in audio or audio and video format (depending on participants’ preferences), and participants will always be asked for their permission before recording. If participants wish, interviews may also be conducted in person at their homes or wherever they deem appropriate.

The use of online interviews may limit the observation of non-verbal communication. To minimize this limitation, video-based interviews will be prioritized whenever possible, and researchers will pay close attention to verbal and paralinguistic cues. In addition, detailed field notes will be taken during and immediately after the interviews to capture contextual and interactional aspects that may not be fully reflected in the recordings.

A pilot interview will be conducted to refine the interview guide, estimate the duration, and ensure the clarity and relevance of the questions. Based on this, interviews are expected to last approximately 40–80 min.

After the interviews have been conducted, they will be transcribed verbatim for subsequent analysis, working only with the text transcripts and the researcher’s field notes. The transcripts and field notes will be anonymized by removing all possible names or identifying information about the participants that may appear in the course of the interview.

The assessment of participants in the QUAN design will be conducted by licensed occupational therapists with more than two years of professional experience in the treatment of individuals with Alzheimer’s disease and in the provision of support and interventions for their families and caregivers. Similarly, the interviews included in the QUAL design, as well as the subsequent data analysis, will be carried out by researchers specialized in qualitative inquiry, each with over five years of experience in this field.

A Data Monitoring Committee (DMC) was not established for this study. Given its parallel convergent mixed-methods design—comprising a cross-sectional observational study and a qualitative multiple case study—and the absence of any pharmacological or high-risk intervention, an independent DMC was not considered necessary. Participant safety, data quality, and adherence to the study procedures will be monitored continuously by the principal investigator and the research team. This approach is consistent with SPIRIT 2013 and ICH-GCP (E6 R2) guidelines, which recommend DMC oversight primarily for multicenter trials or studies involving substantial risk [21]. Any adverse events, unexpected issues, or protocol deviations will be documented and reported to the Research Ethics Committee of Universidad Rey Juan Carlos in accordance with institutional and ethical requirements.

### 2.5. Data Processing

This study involves the processing of personal data, and researchers will therefore guarantee confidentiality at all times. Any personal data that could lead to identification will be removed from the interview.

After inclusion in the study, all participants’ names will be replaced by an alphanumeric code to maintain anonymity. Only the principal investigator will have access to this list. Participants’ names or personal data will never be used when presenting the study results.

The data will be stored in a Microsoft OneDrive folder that only the principal investigator of the study will have access to. QUAL data triangulation will be carried out by three researchers who are part of the research team.

### 2.6. Data Analysis

For the analysis of QUAL data, an inductive qualitative content analysis will be conducted [47,48,49]. This analytical approach is consistent with the case study design, as it allows an in-depth exploration of participants’ experiences within their real-life context, from an interpretive perspective aimed at understanding the meanings attributed to caregiving and bereavement. This approach enables the identification of both manifest (semantic) and latent content, allowing for descriptive and interpretative analyses [48,49].

Interviews will be transcribed verbatim and analysed alongside field notes collected during data collection [50]. The analysis will begin by identifying meaningful units (codes) from the most descriptive content, which will then be grouped into categories based on shared meanings. These categories will be organized to describe participants’ experiences within their specific contexts. Subsequently, latent content will be analysed to generate themes that provide a deeper understanding of participants’ perspectives [48,49].

The analysis will be carried out independently by each researcher for each interview. Subsequently, joint team meetings will be held to compare, refine, and integrate the coding and thematic structure. In case of discrepancies, consensus will be reached among the research team members. The results will be organized into a shared analysis matrix, which will support the identification and definition of final themes. MAXQDA software (version 26) will be used to facilitate data organization and coding. Sampling will follow a non-probabilistic purposive strategy based on maximum variation, in order to capture a wide range of caregiving experiences across different family roles and caregiving contexts. In addition, a snowball sampling technique will be used to facilitate access to participants, particularly in the subgroup of caregivers who have experienced bereavement. Data collection and analysis will be conducted iteratively until data saturation is reached, defined as the point at which no new themes or relevant information emerge within the cases analysed.

For the analysis of QUAN data, both descriptive and inferential statistical analyses will be performed. Descriptive statistics will be used to summarize the characteristics of the sample and the main study variables. The normality of the data will be assessed using histograms, normal Q-Q plots, and the Shapiro–Wilk test. Continuous variables will be described using mean and standard deviation or median and interquartile range, as appropriate, while categorical variables will be presented as frequencies and percentages. Inferential analyses will be conducted to explore potential differences between caregiving contexts (institutionalized care versus home care) and between family roles (husband/wife, son/daughter, grandson/granddaughter). Group comparisons will be performed using parametric tests (e.g., one-way ANOVA) or non-parametric alternatives (e.g., Kruskal–Wallis test), depending on data distribution and homogeneity of variance. Where appropriate, post hoc analyses will be conducted. Given the expected size of some subgroups, analyses involving subgroup comparisons will be interpreted with caution and considered exploratory. Statistical analyses will be performed using SPSS software (version 27).

The data will be integrated using a convergent parallel mixed-methods design, in which the QUAN and QUAL components will be collected and analyzed simultaneously [36]. Integration will take place during the interpretation phase by comparing and contrasting quantitative and qualitative findings to identify convergence, complementarity, or divergence between results. This process will enable the development of joint meta-inferences and a more comprehensive understanding of caregivers’ experiences. The results will be presented through integrated narratives and joint displays.

### 2.7. Procedure for Integrating Quantitative and Qualitative Data

Data integration will be carried out using a mixed concurrent design, in which QUAN and QUAL data collection and analysis will be performed simultaneously [36]. The integration process will consist of interpreting and presenting the findings through joint narratives and joint displays [51,52,53].

Once the independent analysis of each component has been completed, the researchers will jointly integrate the results. The findings from the QUAN and QUAL phases will be presented together in the same section, supported by a table illustrating the integration of the data.

Integration will follow a side-by-side comparison approach, in which quantitative results (e.g., caregiving burden, occupational balance, and quality of life scores) will be aligned with qualitative themes derived from participants’ narratives (e.g., perceived role changes, daily life impact, and experiences of grief). This approach is commonly used in convergent mixed-methods designs to directly compare and relate findings from both datasets [54,55]. This process will allow the identification of areas of convergence (agreement between quantitative and qualitative findings), complementarity (where qualitative data help to explain or expand quantitative results), and divergence (inconsistencies between datasets), as described in mixed-methods integration frameworks [51,52,53].

The integration of both datasets will support the development of meta-inferences, contributing to a more comprehensive understanding of caregivers’ occupational lives and well-being [54,55].

### 2.8. Rigor Criteria

The criteria established by Guba and Lincoln [28,56,57] will be followed to ensure the following: (a) Credibility: triangulation between researchers will be used through meetings during the analysis to identify and compare the themes and results obtained, the use of various data collection instruments (interviews, photographs, focus groups), and validation by the participants; (b) Transferability: detailed descriptions of the study design will be provided; (c) Dependability: external audits will be conducted by a researcher outside the team who will evaluate the research protocol; and (d) Confirmability: reflexivity will be applied through the writing and recording of reflective reports at different stages of the study, especially during data collection and analysis, as well as in ethical considerations.

Reflexivity will be addressed by maintaining reflexive journals throughout the research process, in which researchers will document their assumptions, decisions, and potential influences on data collection and analysis. These reflections will be discussed during team meetings to enhance transparency and minimize potential bias [28,57].

## 3. Discussion

This protocol describes a mixed-methods study designed to explore and understand the life experiences and grieving process of caregivers of people with AD. This project addresses a significant gap in the current literature by integrating quantitative and qualitative data to provide a comprehensive and in-depth understanding of the impact that caregiving and subsequent loss have on the physical, emotional, and social well-being of caregivers.

The majority of previous research has separately analyzed the burden associated with caregiving during the course of the disease and the psychological impact of grief after the death of a family member [10,11]. However, recent evidence suggests that both processes are part of a continuum of experience, in which emotional, social, and occupational transformations begin during the caregiving stage and continue beyond the death of the loved one [13]. By adopting a concurrent mixed design, this study will allow both stages to be examined simultaneously, promoting a more integrated understanding of how caregivers adapt and reconstruct the meaning of their experience over time.

One of the main contributions expected from this study is the construction of a comprehensive model that shows the relationship between the burden of care, anticipatory grief, and subsequent grief as a continuum in the lives of caregivers of people with AD. Recent research has shown that caregivers experience emotional effects even before death, a phenomenon known as anticipatory grief, which manifests itself with symptoms compatible with anxiety, emotional loss, and increased physical burden [58,59]. Furthermore, recent studies on adaptive grief in dementia caregivers suggest that not all grief processes are related to pathological suffering, but that some present adaptive mechanisms that may differ depending on personal resources, support networks, and contextual factors [8,59].

Another emerging dimension is the role of the social environment as a modulator of grief and caregiver burden. A recent systematic review highlights that the social context, including family support, community resources, and social networks, plays a key role in shaping caregivers’ experiences and psychological outcomes, influencing both caregiving burden and the experience of loss before and after death [60].

The integration of quantitative and qualitative data will provide a complementary view of the phenomenon. Quantitative data will allow patterns to be identified in relation to the burden of care, quality of life, and emotional well-being, while qualitative accounts will provide a deeper understanding of the subjective meanings and coping strategies that caregivers develop throughout the care and grief process. The use of joint displays to combine both sources of information will strengthen the validity and interpretation of the results [26]. In this study, integration will be operationalized through a side-by-side comparison of quantitative and qualitative findings, allowing for a structured interpretation of how both datasets relate to each other. Specifically, quantitative results (e.g., caregiving burden, quality of life, and occupational balance) will be examined alongside qualitative themes derived from participants’ narratives (e.g., perceived role changes, emotional experiences, and meaning-making processes). This approach will enable the identification of convergence, complementarity, and divergence between datasets, providing a more nuanced interpretation of caregivers’ experiences. Through this process, meta-inferences will be generated, offering a comprehensive understanding of how objective indicators and subjective experiences interact across the caregiving and bereavement continuum [54,55].

From a practical perspective, this study is expected to provide useful evidence for the design of support interventions aimed at caregivers, not only during the active stage of caregiving but also in the transition to post-loss adjustment. The results may help healthcare and social workers identify protective factors, promote positive adjustment processes, and reduce the risk of prolonged grief or psychological distress.

This mixed-methods study aims to deepen our understanding of the life experiences and grief of caregivers of people with AD, combining quantitative indicators and qualitative narratives. The goal is to capture the complexity of the caregiving and loss process, offering relevant implications for clinical practice, social policy planning, and the development of comprehensive support programs for family caregivers.

### Study Limitations

This study presents several limitations that should be considered when interpreting the findings. First, participants will be recruited from a single residential care home and a day care center, which may limit the diversity and representativeness of the caregiver population. This recruitment strategy may introduce selection bias, as participants are likely to be linked to formal care services and may not reflect the experiences of caregivers who are not connected to such resources. Although recruitment from multiple centers was considered, it was not feasible due to logistical and resource constraints. Consequently, the generalizability of the findings may be limited, and results should be interpreted with caution when extrapolating to other caregiving contexts or populations.

Second, the subdivision of participants into groups according to caregiving context and family role may result in relatively small subgroup sizes. Although inferential statistical analyses will be conducted, comparisons involving subgroups will be interpreted cautiously and considered exploratory.

Finally, as this is a cross-sectional observational study, causal relationships between variables cannot be established. However, the use of a convergent mixed-methods design is expected to provide a more comprehensive understanding of caregivers’ experiences by integrating quantitative and qualitative data.

## 4. Ethics and Dissemination

### 4.1. Research Ethics Approval

This project was approved by the Research Ethics Committee of the Universidad Rey Juan Carlos (code: 041220246522024), prior to the start of the project. The ethical principles for medical research involving human subjects of the Declaration of Helsinki adopted at the 18th Assembly of the World Medical Association (WMA) (Helsinki, Finland, June 1964) and with the latest version revised at the 64th General Assembly of the WMA held in Fortaleza (Brazil) in October 2013 were followed. In addition, they comply with the legislation in force in our country, including Law 14/2007 on Biomedical Research and Royal Decree 223/2004.

### 4.2. Dissemination Policy

The results of this study will be disseminated through publication in high-impact, peer-reviewed journals and presentations at leading national and international scientific conferences to maximize visibility and impact within the research community.

### 4.3. Consent or Assent

In this study, written informed consent will be obtained from each resident or their legal guardians/family members. Moreover, participants will have the option to withdraw or revoke their informed consent at any stage of the study.

### 4.4. Risks and Benefits

Participation in this study is considered to involve minimal risk. However, given that the study explores caregiving experiences and includes a subgroup of participants who have experienced the loss of a relative with AD, there is a potential risk of emotional distress during the interviews, particularly when discussing sensitive topics such as caregiving burden, anticipatory grief, and bereavement.

To minimize this risk, interviews will be conducted by trained researchers with experience in qualitative research and communication skills. Participants will be informed in advance about the nature of the topics to be discussed and will be reminded that they may decline to answer any question or withdraw from the study at any time without any consequences. During the interviews, researchers will monitor participants’ emotional responses and will pause or stop the interview if signs of significant distress are observed.

Regarding participants who have experienced bereavement, only individuals who express willingness to discuss their experience and who do not present evident signs of severe emotional vulnerability at the time of recruitment will be included.

The potential benefits of the study include contributing to a better understanding of caregivers’ experiences, which may help to improve support strategies and healthcare interventions for caregivers of people with AD. Although participants may not receive direct personal benefits, some individuals may find participation meaningful, as it provides an opportunity to reflect on and share their experiences.

### 4.5. Trial Registration

- Name of registry: Understanding the Lived Experience and Bereavement of Caregivers of People With Alzheimer’s Disease (ALCARE)
- ClinicalTrials.gov registration identifying number: NCT07251738.
- Date of registration: November 2025.
- Type of registration: anticipated.
- Protocol version: v2.

## Figures and Tables

**Table 1:** Semi-structured question guide, Group 1.

Area Under Study	Questions	Justification
Diagnosis of AD	What was the process like leading up to your family member’s AD diagnosis?	To explore participants’ initial experiences and emotional responses at the time of diagnosis, as well as the beginning of the caregiving trajectory.
	How did you feel when your family member received the diagnosis?	
Caring for someone with AD before they moved to a residential care home	What was your daily routine like when caring for your family member?	To explore caregiving routines, emotional experiences, occupational balance, and perceived quality of life, as well as decision-making processes and anticipatory grief associated with role transitions.
	What barriers and facilitators did you encounter on a daily basis?	
	Do you think that caring for your family member on a daily basis had an impact on your life?	
	How would you describe your state of mind while caring for your family member?	
	How would you describe your quality of life while caring for your family member?	
	Do you feel that you have stopped doing activities that you used to do? Which ones?	
	How did you come to the decision to place your relative in a nursing home to continue their care?	
Caring for someone with AD who lives in a residential care home	How does your family member living in a nursing home impact your day?	To examine changes in daily life, roles, and occupational balance after institutionalization, as well as adaptation to new caregiving contexts.
	Has your daily life changed since your family member was admitted to the nursing home?	
	What barriers and facilitators do you encounter now that your family member is living in a residential care home?	
Closing Question	What else would you like to share that we have not yet discussed?	To allow participants to introduce additional relevant experiences or perspectives not covered in the interview.

**Table 2:** Semi-structured question guide, Group 2.

Area Under Study	Questions	Justification
Diagnosis of AD	What was the process like leading up to your family member’s AD diagnosis?	To explore initial experiences and emotional responses at the onset of the disease.
	How did you feel when your family member received the diagnosis?	
Caring for someone with AD at home	What was your daily routine like when caring for your family member?	To examine caregiving experiences at home, including routines, emotional impact, occupational balance, and quality of life, as well as decision-making processes and anticipatory grief related to changes in care context.
	What barriers and facilitators did you encounter on a daily basis?	
	Do you think that caring for your family member on a daily basis had an impact on your life?	
	How would you describe your state of mind while caring for your family member?	
	How would you describe your quality of life while caring for your family member?	
	Do you feel that you have stopped doing activities that you used to do? Which ones?	
	How did you come to the decision to take your relative to a day center in a nursing home to continue their care?	
Closing Question	What else would you like to share that we have not yet discussed?	To allow participants to introduce additional relevant experiences or perspectives.

**Table 3:** Semi-structured question guide, Group 3.

Area Under Study	Questions	Justification
Diagnosis of AD	What was the process like leading up to your family member’s AD diagnosis?	To explore early experiences and emotional responses at the beginning of the caregiving process.
	How did you feel when your family member received the diagnosis?	
Caring for someone with AD	What was your daily routine like when caring for your family member?	To explore caregiving experiences, including routines, emotional burden, occupational balance, and perceived quality of life, as well as anticipatory grief and role transitions during the caregiving period.
	What barriers and facilitators did you encounter on a daily basis?	
	Do you think that caring for your family member on a daily basis had an impact on your life?	
	How would you describe your state of mind while caring for your family member?	
	How would you describe your quality of life while caring for your family member?	
	Do you feel that you had stopped doing activities that you used to do? Which ones?	
	How did you come to the decision to place your relative in a nursing home to continue their care?	
Grief/Mourning	How have you been since the death of your family member?	To explore bereavement experiences, including emotional impact, meaning-making, changes in identity and daily life, and adaptation following loss.
	What has the loss of your family member meant to you?	
	To what extent do you think your life has changed since your loss? And in what ways?	
	Do you think your quality of life has changed now compared to when you were caring for your relative?	
	What lessons did you learn from caring for your family member?	
	How do you envision your life moving forward?	
Closing Question	What else would you like to share that we have not yet discussed?	To allow participants to introduce additional reflections, including aspects of grief, meaning, or unmet needs.

## Data Availability

No new data were created or analyzed in this study. Data sharing is not applicable to this article.

## Supplementary Materials

The following supporting information can be downloaded at: https://www.mdpi.com/article/10.3390/healthcare14070899/s1, Table S1: SPIRIT checklist and SPIRIT Timeline. Ref. [61] was cited in Supplementary Materials.

## Abbreviations

The following abbreviations are used in this manuscript:

AD	Alzheimer’s disease
WHO	World Health Organization
ADL	Activities of Daily Living
QUAN	Quantitative study
QUAL	Qualitative study
ZBI	Zarit Burden Interview
OBQ	Occupational Balance Questionnaire
SF-36 QoL-Q	Short Form-36 Health Survey Quality of Life Questionnaire
DMC	Data Monitoring Committee
WMA	World Medical Association
